# Visible-Light-Driven Ag-Modified TiO_2_ Thin Films Anchored on Bamboo Material with Antifungal Memory Activity against *Aspergillus niger*

**DOI:** 10.3390/jof7080592

**Published:** 2021-07-23

**Authors:** Jingpeng Li, Rumin Ma, Zaixing Wu, Sheng He, Yuhe Chen, Ruihua Bai, Jin Wang

**Affiliations:** 1 Key Laboratory of High Efficient Processing of Bamboo of Zhejiang Province, China National Bamboo Research Center, Hangzhou 310012, China; bjfu140524239@163.com (R.M.); jansonwu@126.com (Z.W.); hesheng_cbrc@163.com (S.H.); yuhec@sina.com (Y.C.); oscar_bai168@aliyun.com (R.B.); 2 Zhejiang Provincial Key Lab of Biological and Chemical Utilizing of Forest Resources, Zhejiang Academy of Forestry, Hangzhou 310023, China

**Keywords:** bamboo, Ag/TiO_2_, visible light photoactivity, antifungal activity, energy storage

## Abstract

A round-the-clock photocatalyst with energy-storage ability has piqued the interest of researchers for removing microbial contaminants from indoor environments. This work presents a moderate round-the-clock method for inhibiting the growth of fungus spores on bamboo materials using Ag-modified TiO_2_ thin films. Photoactivated antifungal coating with catalytic memory activity was assembled on a hydrophilic bamboo by first anchoring anatase TiO_2_ thin films (TB) via hydrogen bonding and then decorating them with Ag nanoparticles (ATB) via electrostatic interactions. Antifungal test results show that the Ag/TiO_2_ composite films grown on the bamboo surface produced a synergistic antifungal mechanism under both light and dark conditions. Interestingly, post-illumination catalytic memory was observed for ATB, as demonstrated by the inhibition of *Aspergillus niger* (*A. niger*) spores, in the dark after visible light was removed, which could be attributed to the transfer of photoexcited electrons from TiO_2_ to Ag, their trapping on Ag under visible-light illumination, and their release in the dark after visible light was removed. The mechanism study revealed that the immobilized Ag nanoparticles served the role of “killing two birds with one stone”: increasing visible-light absorption through surface plasmon resonance, preventing photogenerated electron–hole recombination by trapping electrons, and contributing to the generation of ●O_2_^−^and ●OH. This discovery creates a pathway for the continuous removal of indoor air pollutants such as volatile organic compounds, bacteria, and fungus in the day and night time.

## 1. Introduction

Indoor air pollution has been described as the most significant environmental cause of death globally, accounting for an estimated 3.8–4.3 million premature deaths each year over the past decade [1]. In major cities around the world, people spend more than 90% of their time in confined indoor environments. There is evidence that short-term exposure of human subjects to air pollution may exacerbate asthma and lead to hospitalizations, whereas long-term exposure to air pollution is repeatedly associated with a higher incidence of cardiovascular and respiratory diseases, birth defects, and neurodegenerative disorders. Fungi are ubiquitous and are a serious threat to public health in indoor environments [2].

Fungi can grow on almost all natural and synthetic materials, especially if they are hygroscopic or wet. As common indoor building materials, inorganic [3], wood-based [4], and bamboo-based materials [5] could serve as good growth substrates for fungi. In recent years, bamboo has received considerable attention because of its high strength, fast growth, renewability, and carbon sequestration potential [6]. All types of bamboo products have been developed and used in interior construction, decoration, and furniture materials worldwide. Nevertheless, bamboo is highly vulnerable to fungal attacks, especially during the rainy season. Therefore, efficient and environmentally friendly methods for fungi inhibition are highly desirable.

Semiconductor photocatalysis has been considered one of the most promising technologies for environmental purification, as additional chemical compounds such as strong oxidants are not introduced into the environment, and energy consumption is much lower than that of other advanced oxidation technologies [7]. Among semiconductors, TiO_2_ has proven to be the most suitable photocatalyst because of its abundance, chemical stability, nontoxicity, and low cost [8]. Nevertheless, TiO_2_ can harvest only ultraviolet (UV) light and has a high recombination rate of electron–hole pairs, leading to low photocatalytic efficiency [9]. The key issue for TiO_2_-based photocatalysts is tuning their photoactive range toward the visible light region (λ > 400 nm); thus, more solar energy can be used. To overcome this problem, we recently conducted studies to enhance photocatalytic efficiency and antifungal activities, such as decorating ZnO nanoparticles (NPs) on TiO_2_ film or doping Fe^3+^ into TiO_2_ films [10,11].

Photocatalysis requires a continuous light source to facilitate redox reactions. From the practical application perspective, we do not want antifungal photocatalysts to be constantly exposed to light. Presently, increasing efforts are being made to develop photocatalysts for photocatalytic reactions in both light and dark conditions, termed “round-the-clock photocatalysis” or “memory catalysis” [12]. Energy-storage substances such as carbon nanotubes [13], C_3_N_4_ [14], Se [15], Bi [16], WO_3_ [17] and MoO_3_ [18] have been developed for catalytic memory reactions. In addition to these nanomaterials, Ag NPs can also store electrons because of their capacitive activity [12]. The capacitive nature of Ag NPs impedes the charge transfer of trapped electrons out of their surface. Kamat et al. [19] suggested that electron storage depends on the amount of Ag deposited on TiO_2_ NPs. Choi et al. [20] investigated a sequential photocatalysis-dark reaction, where organic pollutants were degraded on Ag/TiO_2_ under UV irradiation and the storage of electrons in Ag/TiO_2_, which were then used to reduce Cr(VI) in the post-irradiation period. Liu et al. [21] and Jiao et al. [22] presented a new strategy to improve the catalytic memory activity of Ag/TiO_2_ for organic contaminant removal under UV light. In addition, Ag nanomaterials are widely used as antimicrobials [23]. In addition to their toxicity, they could produce a synergistic antibacterial effect with other nanomaterials such as TiO_2_ [24]. Chen et al. [25] also showed that the size of the Ag nanostructure is a critical factor in antibacterial capacity. Despite research in this area, few studies have focused on the use of energy-storing photocatalysts for mildew control, let alone under visible light conditions.

In this study, a photoactivated antifungal coating with catalytic memory activity was assembled on the surface of a hydrophilic bamboo by first anchoring anatase TiO_2_ thin films and then decorating Ag NPs. Different characterization methods were used to analyze the structural and optical properties of Ag-modified TiO_2_ thin films grown on the bamboo surface. The Ag/TiO_2_ composite films grown on the bamboo surface produced a synergistic antifungal mechanism under both light and dark conditions. Remarkably, post-illumination catalytic memory was observed for ATB in the dark after visible light was removed, as demonstrated by the inhibition of *A. niger* spores. The mechanisms involved in the antifungal processes of Ag/TiO_2_ under both dark and visible-light conditions are discussed and proposed.

## 2. Materials and Methods

### 2.1. Materials

Air-dried moso bamboo (*Phyllostachys edulis* (Carr.) J.Houz.) specimens with dimensions of 50 mm (longitudinal) × 20 mm (tangential) × 5 mm (radial) were purchased from Zhejiang YoYu Corporation. All chemicals used in the experiments were of analytical reagent grade. Potato dextrose agar (PDA; 1 L of water, 6 g potato, 20 g dextrose, and 20 g agar, pH = 5.6) was obtained from Qingdao Hope Bio-Technology Co., Ltd. Deionized water was prepared using a Milli-Q Advantage A10 water purification system (Millipore, Billerica, MA, USA) and used throughout all experiments.

### 2.2. Preparation of Ag-Modified TiO_2_ Thin Films on the Bamboo Surface

The TiO_2_ thin films were synthesized on the bamboo surface via a modified procedure according to our previous work [26]. In particular, the mixed solution of (NH_4_)_2_TiF_6_ and H_3_BO_3_ was sufficiently transferred into a 50 mL Teflon-lined autoclave without pH adjustment and heated at 90 °C for 4 h in an oven. A TiO_2_ thin-film-coated bamboo sample was denoted as TB. Loading of Ag NPs on the surface of TB was achieved in the dark with a simple and rapid silver mirror method. Ammonia solution (25–28%) was added dropwise into 100 mL of AgNO_3_ solution until the brown precipitate was dissolved. Then, a TB sample was submerged in the silver ammonia solution under feeble stirring for 1 h. Subsequently, the sample was transferred into a 0.2 M glucose solution until [Ag(NH_3_)_2_]^+^ ions absorbed on the TB sample were completely reduced. Finally, the samples were repeatedly washed with deionized water and dried at 50 °C for 24 h in an oven. The Ag-NP-decorated TB samples were denoted as ATB-*x,* with *x* representing the solution concentration (5, 10, 30, 50, and 200 mM) of AgNO_3_ as one of the raw materials. For example, ATB-10 indicates that the solution concentration of AgNO_3_ was 10 mM, which was used to load the Ag NPs on the TB surface. Ag/bamboo (AB) samples were also prepared following the above-mentioned procedure.

### 2.3. Characterization

The crystal structures of the samples were determined by X-ray diffraction (XRD, Bruker D8 Advance, Germany) using Cu Kα radiation (λ = 1.5418 Å) and scanning over a 2*θ* range of 10° to 80°. The surface morphologies of the samples were observed using a scanning electron microscope (SEM, Hitachi S3400, Tokyo, Japan) equipped with an energy-dispersive X-ray spectroscopy (EDS) system. The UV–visible (UV–Vis) absorption spectra of the samples were obtained using a Scan UV–Vis spectrophotometer (Hitachi U-3900, Tokyo, Japan). The spectra were recorded in the range of 200–800 nm at room temperature in air. The compositions of the samples were inferred following X-ray photoelectron spectroscopy (XPS, Thermo ESCALAB 250Xi, Waltham, MA, USA) results, which were obtained using an ESCALab MKII X-ray photoelectron spectrometer with Al Kα X-ray radiation as the excitation source. The photoluminescence spectra (PL) of the samples were obtained using the Edinburgh FLS 980 (Edinburgh, UK) fluorescence spectrometer. The electron spin resonance (ESR) signals of radicals trapped by 5,5-dimethyl-1-pyrroline *N*-oxide (DMPO) were detected at ambient temperature using a Bruker (E580, Rheinstetten, Germany) spectrometer under visible-light irradiation (*λ* > 400 nm).

### 2.4. Antifungal Test

Antifungal tests of the as-prepared samples were conducted according to the Chinese Standard GB/T 18261-2013, with some modifications. For all experiments, *A. niger* was used, which is a common fungus that infects bamboo. *A. niger* spores were obtained from BeNa Culture Collection (BNCC, Beijing, China) and were activated before use. After being activated, the *A. niger* spores (approximately 1 × 10^6^ CFU/mL (CFU = colony-forming unit)) were inoculated on each PDA plate at 28 °C and 90% relative humidity for 7 days until sporulation. Prior to inoculation, the as-prepared samples and U-shaped glass rod were sterilized using a steam sterilizer at 121 °C and 0.1 MPa for 30 min using an autoclave (SANYO, MLS-3750, Osaka, Japan). A sterilized U-shape glass rod (4 mm in diameter) was placed on the PDA substrate, which was covered with mycelium, and two specimens were placed separately on the glass rod. Subsequently, the dishes were placed in a climate chamber (Boxun, BIC-400, Shanghai, China), where temperature and relative humidity were fixed at 28 °C and 90%, respectively. The tests were conducted for 28 days.

The as-prepared samples, including the original bamboo, TB, AB-10, AB-30, ATB-5, ATB-10, ATB-30, and ATB-200, were used for the antifungal tests with and without visible-light irradiation (Figure S1). One group was analyzed under visible-light radiation (Philips TLD30W/54) for 6 h every day and then the light source was turned off, whereas the other was analyzed in the dark. All experiments were sextuplicated.

## 3. Results and Discussion

### 3.1. Structural Investigations

**Figure 1:**
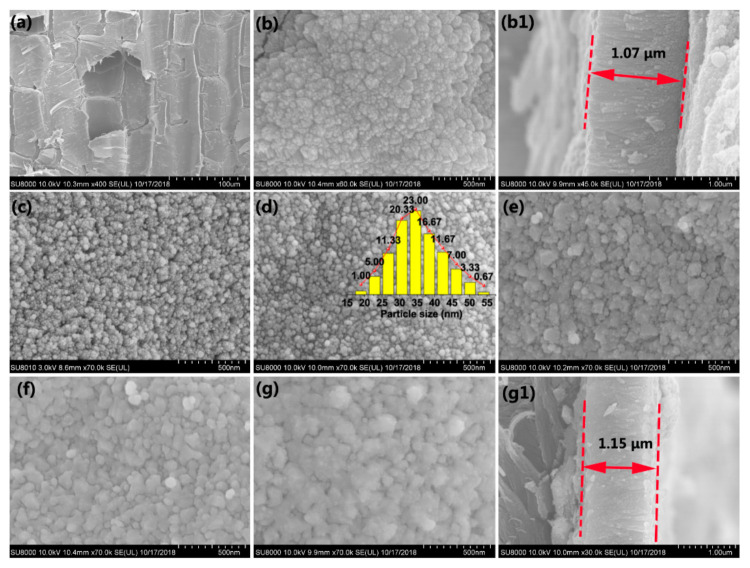
SEM images of (**a**) unvarnished bamboo, (**b**) TB and its corresponding cross-sectional profile (**b1**), (**c**) ATB-5, (**d**) ATB-10 and the size distribution of Ag nanocrystals (inset), (**e**) ATB-30, (**f**) ATB-50, and (**g**) ATB-200 and its corresponding cross-sectional profile (**g1**). TB: TiO_2_/bamboo, ATB-*x*: the Ag-NP-decorated TiO_2_/bamboo samples were denoted as ATB-*x*, with *x* representing the solution concentration (5, 10, 30, 50, and 200 mM) of AgNO_3_ as one of the raw materials.

Figure 1a presents the SEM image of the original bamboo, which comprises numerous sizable parenchyma cells. No other substances were observed on the bamboo surfaces, except the microstructure of the bamboo. After the first step, the TiO_2_ thin films with an average thickness of 1.07 μm were self-aggregated by homogeneous TiO_2_ NPs on the bamboo surface (Figure 1b,(b1)). Figure 1c–g display the SEM images of the as-prepared samples with different concentrations of AgNO_3_. As shown in Figure 1c, few Ag NPs appear for an AgNO_3_ concentration of 0.005 M. As the AgNO_3_ concentration increased from 0.005 to 0.01 M (Figure 1d), the nanosized Ag particles with an average size of ~35 nm were uniformly deposited on the TiO_2_ thin films. As shown in Figure 1e, as the AgNO_3_ concentration increased to 0.03 M, the particles were self-aggregated together, increasing the average diameters of the Ag NPs accordingly. Most of the uniform Ag NPs gradually vanished, and the particles became denser and even dissolved one another, forming Ag thin films on the TiO_2_ surface (Figure 1g). The average thickness of composite thin films is approximately 1.15 μm (Figure 1(g1)).

**Figure 2:**
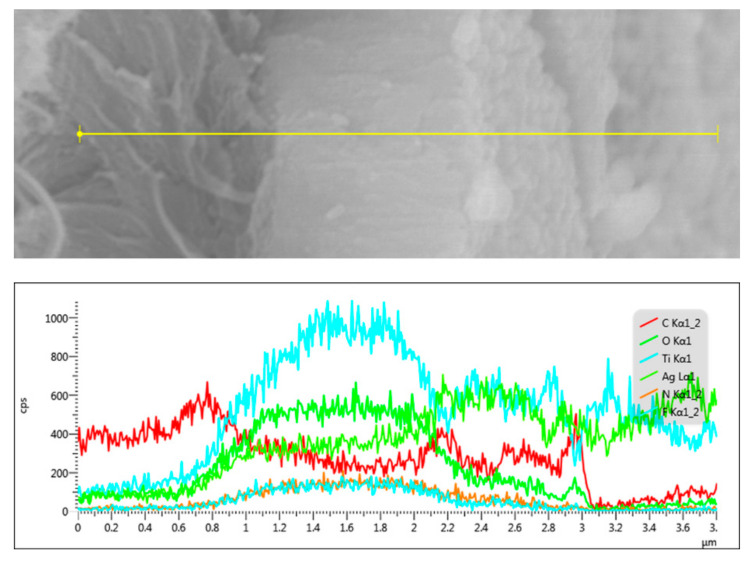
SEM in the line-scanning mode and the element distribution for a cross-sectional profile of ATB-10. ATB-10: Ag/TiO_2_/bamboo; the solution concentration of AgNO_3_ used is 10 mM.

The structure of the ATB-10 sample was further studied by EDS. EDS results confirmed the presence of Ti, Ag, O, F, and C, whereas elemental mapping revealed that the Ti and Ag components were broadly and densely dispersed over the entire sample surface (Figure S2). Figure 2 shows the relative intensity of each element in the EDS spectrum measured along the thickness direction (yellow line). The signals of C, Ti, and Ag at different positions indicated that the Ag-modified TiO_2_ composite thin films were successfully anchored to the bamboo surface.

**Figure 3:**
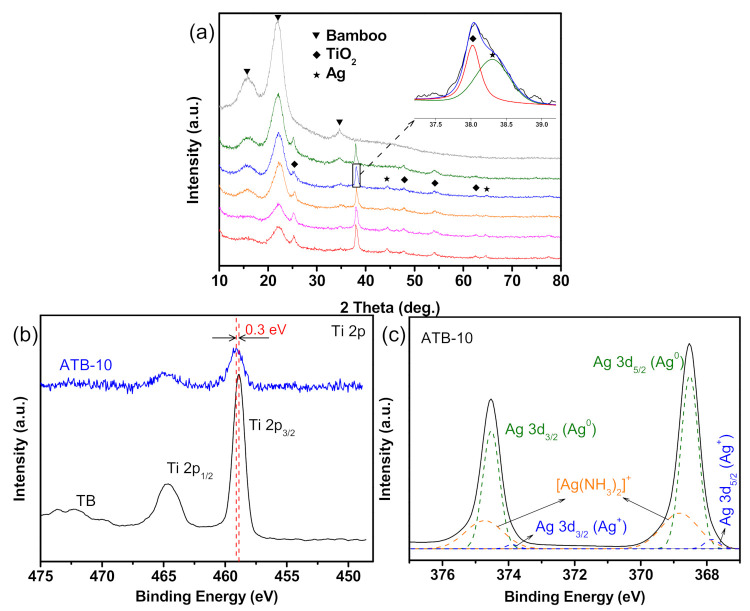
(**a**) XRD patterns from top to bottom are those of original bamboo, ATB-5, ATB-10, ATB-30, ATB-50, and ATB-200. The inset shows a part of the amplification of the XRD pattern (ATB-10). The high-resolution XPS spectra of (**b**) Ti 2p and (**c**) Ag 3d. ATB-*x*: the Ag-NP-decorated TiO_2_/bamboo samples were denoted as ATB-*x*, with *x* representing the solution concentration (5, 10, 30, 50, and 200 mM) of AgNO_3_ as one of the raw materials.

**Figure 4:**
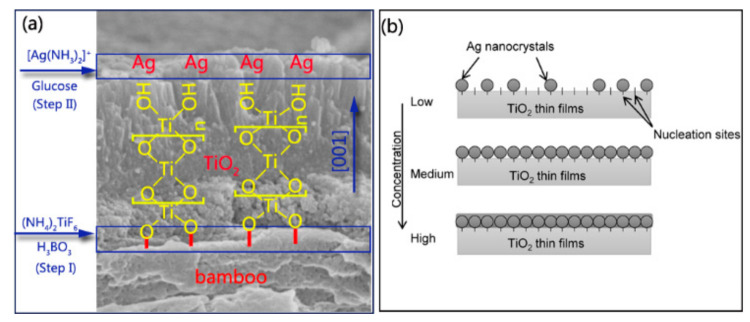
(**a**) Mechanism and process of nanosized Ag-modified TiO_2_ thin films anchored to the bamboo surface; (**b**) schematic representation of the mineralization model proposed for the deposition of Ag nanocrystals on TiO_2_ thin films with low, medium, and high densities of [Ag(NH_3_)_2_]^+^ solution concentration.

The detailed crystal structures and chemical composition of the as-prepared samples were analyzed by XRD and XPS. As shown in Figure 3a, all samples exhibited similar diffraction peaks at approximately 16°, 22°, and 35°, which can be ascribed to the crystalline cellulose in bamboo. The samples all exhibited a typical anatase TiO_2_ phase (JCPDS NO.71-1167), except the original bamboo. Additional diffraction peaks appeared at 38.1°, 44.4°, and 64.6°, which were assigned to the (111), (200), and (220) lattice planes of Ag, respectively [27]. No other characteristic diffraction peaks for impurities were observed in the pattern. However, the Ag diffraction peaks of the ATB-5 (green) sample could not be observed because of the relatively small amount and high dispersion of Ag metal. Notably, increasing the AgNO_3_ concentrations from 0.01 to 0.2 M had no discernible effect on the diffraction peak intensity of the Ag metal phase. However, the diffraction peak intensity of crystalline cellulose decreased, suggesting that more Ag NPs were self-aggregated together, forming Ag thin films on the TB surface. This result is consistent with the SEM analysis, which also supported the conjecture of growth mechanism of Ag NPs on the TB surface, as shown in Figure 4b.

Research has previously suggested that only metallic Ag NPs have electron-storage ability. The chemical compositions and valence of Ag were further confirmed by XPS analysis. As shown in Figure S3, the survey spectra of ATB-10 revealed the existence of Ag, O, Ti, F, and C, which was consistent with the EDS results. As shown in Figure 3b, the XPS result of TB shows the core levels of Ti 2p_1/2_ and Ti 2p_3/2_ to be approximately at 464.6 and 458.9 eV, respectively, which was assigned to the Ti^4+^ in anatase TiO_2_. However, the Ti 2p binding energy of ATB-10 is slightly shifted from 458.9 to 459.2 eV compared with that of TB. This is because the Fermi level of Ag is lower than that of TiO_2_, so the conduction-band electrons of TiO_2_ may be transferred to the Ag deposited on the surface of TiO_2_, which decreases the outer electron cloud density of Ti ions [28]. Figure 3c shows the high-resolution XPS scans over the Ag 3d peak. The main peaks at 368.5 and 374.5 eV were ascribed to Ag metal, while the binding energies at 367.8 and 373.8 eV were attributed to Ag_2_O. The two peaks detected at 368.8 and 374.7 eV could be attributed to Ag(NH_3_)_2_^+^ ions [29]. This observation and XRD analysis results suggested that a small portion of Ag on the NP surface was oxidized to Ag_2_O during sample drying and handling under normal ambient conditions, and the amount of Ag_2_O was too small to be detected by XRD. Many researchers have reported that a small amount of Ag_2_O on the Ag NP surface could enhance its stability [30].

### 3.2. Formation Mechanism of ATB Samples

Bamboo is hydrophilic, with plentiful active hydroxyl groups, and the hydroxyl groups in a bamboo substrate can react with certain metal oxides such as ZnO [31], TiO_2_ [26], γ-Fe_2_O_3_ [32], and Cu_2_O [33]. This method uses the hydrolysis of a solution containing TiF_6_^2−^ in the presence of H_3_BO_3_ as a fluoride scavenger. The fabrication of TiO_2_ thin films on the bamboo surface was accomplished by heterogeneous nucleation and homogeneous growth. For the initial heterogeneous nucleation on the bamboo surface, the existence of plentiful R–OH groups as active sites promoted the formation of R–O–Ti linkages between the bamboo surface and TiO_2_ particles (Figure 4a).

(bamboo)R–OH + HO–Ti → (bamboo)R–O–Ti + H_2_O (1)

The nucleated TiO_2_ layer on the bamboo substrate could serve as the seed layer to further boost the homogeneous condensation of the TiO_2_ NPs. For the further growth of TiO_2_ NPs, the Ti–OH groups present on the surface of previous TiO_2_ NPs connected with bamboo could continue to act as the active sites for the subsequent particle growth via olation and oxolation, forming Ti–O–Ti linkage (Figure 4a) [34].

Ti–OH + HO–Ti → Ti–O–Ti + H_2_O (2)

From the cross-sectional profile of TiO_2_ thin films, columnar crystal growth in the (001) direction can be seen on the bamboo substrate (Figure S4). This columnar morphology is consistent with the XRD measurement, which showed a significantly enhanced peak of (004) reflection (Figure S5). Previous research has demonstrated that the selective adsorption of anions on specific surfaces parallel to the (001) direction can inhibit crystal growth perpendicular to the (001) direction [34]. In our project, different types of anions, such as F^−^, BO_3_^3−^, BF_4_^−^, and TiF_6_^2−^, were included, which could influence the growth orientation of TiO_2_ crystals. Furthermore, the ζ potential of TiO_2_ particles obtained using this reaction system was also confirmed to be negative owing to the strong adsorption of anions contained in the solution [34]. The XPS results also supported this standpoint because the presence of F^−^ anions on the surface of TB and the F^−^ ions on the TiO_2_ surface could act as the active sites for the subsequent Ag nanocrystal growth (Figure S6).

In step II, when [Ag(NH_3_)_2_]^+^ was introduced, positively charged anions were drawn to a negatively charged TiO_2_ surface covered by F^−^ or OH groups owing to an attractive electrostatic force [35]. The silver mirror reaction generally involves the chemical reduction of the Ag compound into elemental Ag in the solution. The formed Ag subsequently nucleated on the surface of TiO_2_ thin films. Figure 4b illustrates the nucleation mechanism of Ag nanocrystals that can be proposed based on SEM observations (Figure 1c–g). TB surfaces provide a certain number of nucleation sites to synthesize Ag nanocrystals. At low-level concentrations of [Ag(NH_3_)_2_]^+^, the nucleation sites are sufficient to deposit Ag nanocrystals. Ag nanocrystals are uniformly deposited on nucleation sites as the concentration of the precursor solution increases. If a high concentration of the precursor solution is provided, the nucleation sites are insufficient for grafting the Ag nanocrystals, resulting in the formation of Ag thin films coated on the surface of TB, as shown in Figure 1c–g.

### 3.3. Optical Properties

**Figure 5:**
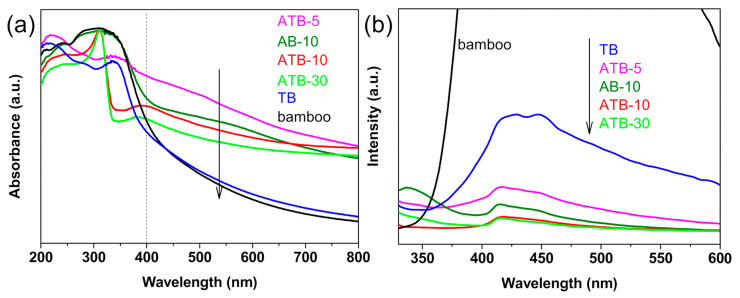
(**a**) UV–Visible DRS and (**b**) PL spectra (excited wavelength: 300 nm) of the original bamboo, TB, AB-10, and ATB prepared in the presence of AgNO_3_: 5, 10, and 30 mM. TB: TiO_2_/bamboo, ATB-*x*: the Ag-NP-decorated TiO_2_/bamboo samples were denoted as ATB-*x*, with *x* representing the solution concentration (5, 10, and 30 mM) of AgNO_3_ as one of the raw materials, AB-10: Ag/ bamboo; the solution concentration of AgNO_3_ used is 10 mM.

Figure 5a presents the UV−Vis absorbance spectra of the original bamboo, TB, AB-10, and ATB prepared in the presence of AgNO_3_: 5, 10, and 30 mM. The original bamboo exhibits strong absorption in the UV region and poor light absorption in the visible-light region from 400 to 800 nm as well as the TB sample. The samples exhibited strong visible-light absorption after the addition of Ag NPs owing to localized surface plasmon resonance. In other words, they react to visible light. Moreover, the smaller the size of Ag NPs, the greater the intensity of light absorption [36]. When comparing ATB-30 with ATB-10, the intensity of visible-light absorption decreased, implying that the Ag NPs began to grow and agglomerate. The results in Figure 5a are consistent with SEM experimental data. The efficiency of plasmon-mediated electron transfer is dominated by the size of the Ag NPs, which plays a critical role in determining the reduction potentials of the electrons transferred to the TiO_2_ conduction band [37].

For semiconductor nanomaterials, the PL spectra are related to the transfer behavior of the photoinduced electrons and holes, so the separation and recombination of photoinduced charge carriers can be reflected. Figure 5b shows the PL spectra of the original bamboo, TB, AB-10, and ATB prepared in the presence of AgNO_3_: 5, 10, and 30 mM. We discovered that the original bamboo had a much higher PL intensity than other samples. Compared with TB, the intensity of the PL signal for the Ag-decorated samples was much lower, indicating that the deposition of Ag reduced the recombination rate of electrons and holes under light irradiation. The PL intensities of these samples varied in the following order: original bamboo > TB > ATB-5 > AB-10 > ATB-10 ≈ ATB-30. This result could be attributed to the existence of Ag NPs decorated on the TiO_2_ thin films, which act as electron trappers to inhibit the recombination of photogenerated electrons and holes and decrease the PL intensity. Generally, the low PL intensity showed a high separation rate of photogenerated electron–hole pairs, resulting in a high photocatalytic activity. Therefore, a lower PL intensity indicates that the ATB samples have higher photocatalytic activities [38].

### 3.4. Antifungal Performance of Ag-Modified TiO_2_ Thin Films

#### 3.4.1. Inhibition of *A. niger* Spores in Darkness

**Figure 6:**
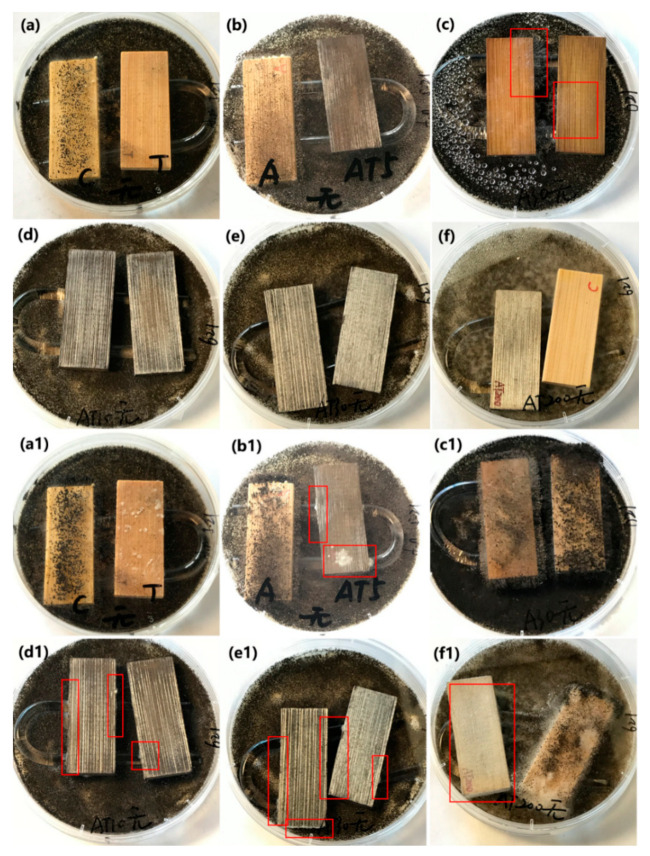
Antifungal properties of (**a**,**a1**) original bamboo (left) and TB (right), (**b**,**b1**) AB-10 (left) and ATB-5 (right), (**c**,**c1**) AB-30, (**d,d1**) ATB-10, (**e**,**e1**) ATB-30, and (**f**,**f1**) ATB-200 (left) and original bamboo (right) to inhibit *A. niger* growth in darkness. We can clearly see the mycelia in the red rectangle. Incubation period: (**a**–**f**) 5 days, (**a1**–**f1**) 28 days. TB: TiO_2_/bamboo, ATB-*x*: the Ag-NP-decorated TiO_2_/bamboo samples were denoted as ATB-*x*, with *x* representing the solution concentration (5, 10, 30, and 200 mM) of AgNO_3_ as one of the raw materials, AB-*x*: the Ag-NP-decorated bamboo samples were denoted as AB-*x*, with *x* representing the solution concentration (10 and 30 mM) of AgNO_3_ as one of the raw materials.

The antifungal activity of the original bamboo, TB, AB-10, AB-30, ATB-5, ATB-10, ATB-30, and ATB-200 and their inhibition ability against *A. niger* spores in the dark are shown in Figure 6. A U-shaped glass rod was used to support the test specimens on the mycelia-covered PDA substrates, preventing their direct contact with the spores. Only five days were required for the mycelia to grow over the entire surface of the original bamboo (Figure 6a, left), indicating that the original bamboo had no resistance to *A. niger*. Peculiarly, mycelia could grow well on the bamboo surface of the AB-10 sample after incubation for five days, even though many Ag NPs were coated on the bamboo surface (Figure 6b, left). Although we increased the concentration of [Ag(NH_3_)_2_]^+^ ions to prepare more Ag NPs on the bamboo surface, the AB-30 could not inhibit the growth of mycelia completely after incubation for five days (Figure 6c). These results indicate that the AB samples had poor resistance to *A. niger*.

Our previous work similarly showed that nanosized Ag-treated bamboo samples have poor resistance to *P. citrinum and T. viride* [35]. In addition, we did not observe any mycelial growth on the surfaces of the TB, ATB-5, ATB-10, ATB-30, and ATB-200 samples after incubation for five days. After incubation for 28 days in the dark (Figure 6(a1–f1)), all samples displayed varying degrees of fungus infection. The surfaces of the original bamboo, AB-10, AB-30, TB, and ATB-200 were almost entirely covered with mycelia in the optical images (Figure 6), indicating that they had no resistance to *A. niger* in the dark. A few mycelia were directly observed on the surfaces of the ATB-5, ATB-10, and ATB-30 samples. Mycelia were mainly found on the side of the samples, especially in ATB-10 and ATB-30 samples. These results indicate that the ATB-5, ATB-10, and ATB-30 samples had a certain degree of resistance to *A. niger* in the dark. These observations suggest that the Ag/TiO_2_ composite produces a synergistic antifungal effect that is unrelated to photoactivity.

#### 3.4.2. Inhibition of *A. niger* Spores under Alternating Visible-Light Irradiation and Dark Conditions

**Figure 7:**
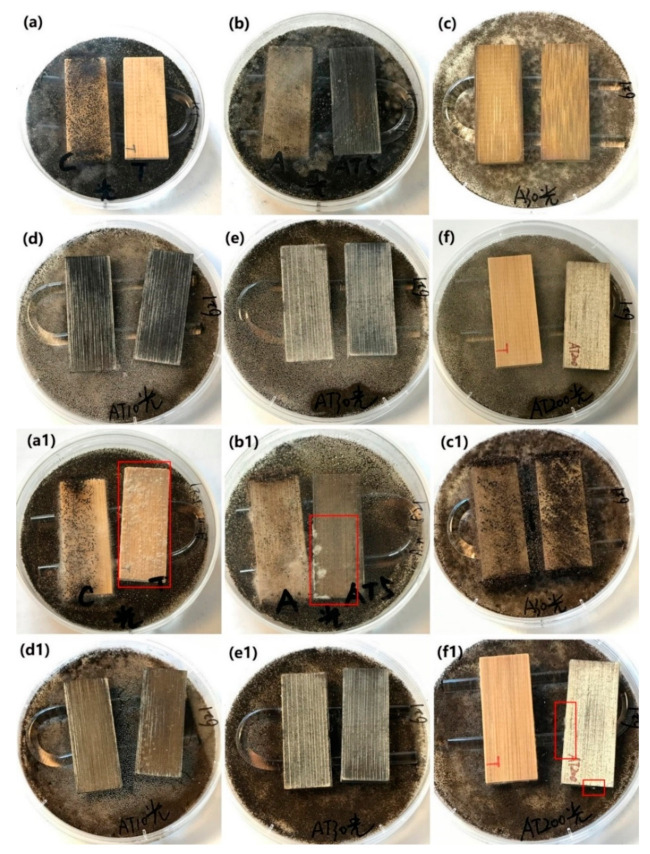
Antifungal properties of (**a**,**a1**) original bamboo (left) and TB (right), (**b**,**b1**) AB-10 (left) and ATB-5 (right), (**c**,**c1**) AB-30, (**d**,**d1**) ATB-10, (**e**,**e1**) ATB-30, and (**f**,**f1**) TB (left) and ATB-200 (right) to inhibit *A. niger* growth under LED light. We can clearly see the mycelia in the red rectangle. Incubation period: (**a**–**f**) 5 days, (**a1**–**f1**) 28 days. TB: TiO_2_/bamboo, ATB-*x*: the Ag-NP-decorated TiO_2_/bamboo samples were denoted as ATB-*x*, with *x* representing the solution concentration (5, 10, 30, and 200 mM) of AgNO_3_ as one of the raw materials, AB-*x*: the Ag-NP-decorated bamboo samples were denoted as AB-*x*, with *x* representing the solution concentration (10 and 30 mM) of AgNO_3_ as one of the raw materials.

The as-prepared samples, including the original bamboo, TB, AB-10, AB-30, ATB-5, ATB-10, ATB-30, and ATB-200 samples, were used for the antifungal test to inhibit *A. niger* spores under visible-light irradiation. The samples were tested under light radiation for 6 h every day, and then the light source was turned off. Figure 7 shows that the original bamboo and AB-10 samples were almost entirely covered with mycelia after incubation for five days, indicating poor resistance to *A. niger* under visible light. However, in addition to the original bamboo and AB-10, the TB and AB-30 samples also failed to inhibit the growth of *A. niger* after incubation for 28 days, even under visible-light irradiation. Optical images showed that their surfaces were almost entirely covered with mycelia (Figure 7(a1,c1)), indicating that they had no resistance to *A. niger*. Multiple fungal clusters were observed on the surface of ATB-5 after incubation for 28 days (Figure 7(b1), right). However, the ATB-10 and ATB-30 samples showed better antifungal activity than other samples, as *A. niger* mycelia failed to cover the entire surface of the samples after incubation for 28 days (Figure 7(d1,e1)). Note that ATB-200 exhibited better antifungal activity for *A. niger* under visible-light irradiation (Figure 7(f1), right) than under dark conditions (Figure 6(f1), left). This may be due to the plasmonic resonance effect of Ag metal under visible-light irradiation, inhibiting the growth of *A. niger* spores [39].

#### 3.4.3. Discussion of the Antifungal Mechanisms

In this work, the hybrid Ag/TiO_2_ films grown on the bamboo surface produced a synergistic antifungal mechanism under both light and dark conditions. According to data from the experiments conducted in the dark, the Ag/TiO_2_ NPs showed more effectiveness at inhibiting *A. niger* growth than pure Ag NPs or TiO_2_ NPs, even though they could not completely inhibit the growth of *A. niger*. The mechanism for the enhanced antimicrobial effect of Ag/TiO_2_ hybrids in the absence of light is still not completely understood. Their enhanced antimicrobial qualities originated from the generation of reactive oxygen species, the release of toxic Ag ions, and cell membrane damage through their contact with the Ag NPs.

Hoek et al. [24] reported that hybrid Ag/TiO_2_ NPs exhibited stronger bactericidal activity than pure Ag and TiO_2_ in the absence of light. The observed synergistic effects under dark conditions were most likely caused by the variation in the dissolution and reprecipitation kinetics and equilibrium between pure Ag NPs and Ag/TiO_2_ NPs. Kim et al. [40] hypothesized that the toxicity of Ag NPs is mainly caused by oxidative stress and is not related to the activity of Ag ions. Perkas et al. [41] proposed that the antibacterial activity of Ag/TiO_2_ composites originates from the presence of reactive oxygen species (ROS) as well as Ag ions on the surface of TiO_2_ in the dark. Chen et al. [25] reported that the antibacterial activity of Ag/TiO_2_ nanocomposites under dark conditions appears to be superior to that of some pure Ag NPs. They suggested that the smaller Ag particle size should account for the higher antibacterial activity of their Ag/TiO_2_. Perkas et al. [41] and Esfandiari et al. [42] both reported a similar observation, noting that the bactericidal capacity depended on the size characteristics of the Ag/TiO_2_ coating. Under similar testing conditions, our previous work showed that TiO_2_ thin films modified by Ag NP (diameter of 2–10 nm) have better antifungal activity for bamboo than those modified by large Ag NPs (diameter of 50–100 nm) [35]. In addition, the antifungal performance of Ag/TiO_2_ nanocomposites was greater than that of AB and TiO_2_/bamboo in the absence of light, indicating that the Ag/TiO_2_ nanocomposite produced a synergistic antifungal effect that was unrelated to photoactivity.

As mentioned above, under dark conditions, the ATB samples could not completely inhibit *A. niger* growth on their surfaces. It is widely considered that photocatalytic microorganism disinfection depends on the interaction between microorganisms and ROS generated from photocatalysts under light illumination, such as ●OH and ●O_2_^−^, which can kill microorganisms [43]. Therefore, we further evaluated the antifungal activity of as-prepared samples to inhibit the growth of *A. niger* under light radiation. From the practical application perspective, photocatalysts should not be constantly exposed to light. Therefore, we attempted to perform our experiment under visible-light irradiation for 6 h every day and then turn off the light source. Interestingly, some of the as-prepared samples could achieve complete antimicrobial activity. The ATB samples exhibited strong visible-light absorption after the addition of Ag NPs owing to the localized surface plasmon resonance. They could generate electron–hole pairs under visible-light irradiation and then migrated to the surface of the catalyst to initiate redox reactions. Most interestingly, the as-prepared ATB samples could store electrons after visible light was removed.

**Figure 8:**
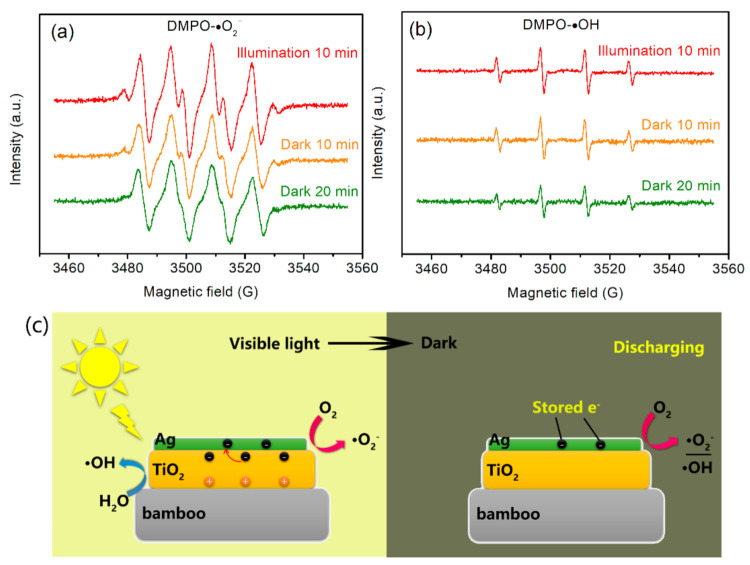
Time evolution of (**a**) DMPO-●O_2_^−^ and (**b**) DMPO-●OH ESR spectra for ATB-10. (**c**) Possible mechanism of the catalytic memory reaction. ATB-10: Ag/TiO_2_/bamboo; the solution concentration of AgNO_3_ used is 10 mM.

Figure 8 presents the ESR spectra of the ATB-10 sample. After 10-min visible-light irradiation, the strong characteristic peak DMPO-●O_2_^−^ signals were observed, which demonstrates the formation of ●O_2_^−^ radicals by ATB-10 under light illumination (Figure 8a). When illumination was turned off, the four peaks associated with DMPO-●O_2_^−^ adducts for ATB-10 could still be distinguished. The intensity of the DMPO-●O_2_^−^ signals was slightly reduced after the sample was kept in the dark for 20 min. This result demonstrates that ●O_2_^−^ could be produced by ATB-10 during a dark discharge process. Similarly, we also verified the formation of ●OH radicals in the dark. The ATB-10 sample exhibited slower decay kinetics of DMPO-●OH adducts after being kept in the dark for 20 min, as shown in Figure 8b. This result indicates that a considerable number of electrons in ATB-10 may remain when illumination is stopped, providing additional ●OH to mitigate the decay of DMPO-●OH, which is consistent with previous work [44]. Based on the experimental data and analysis, a possible mechanism for the memory antifungal activity can be proposed as follows (Figure 8c):

During the photocatalytic disinfection period, excess electrons can be trapped on Ag NPs because of the capacitive nature of Ag nanomaterials. Stored electrons will be released in the dark and subsequently discharged to appropriate electron acceptors, such as O_2_ and H_2_O, to produce the corresponding active free radicals to inhibit the growth of fungi [44]. The combination of photocatalytic disinfection and catalytic memory reaction provides a new pathway for producing novel catalysts to achieve round-the-clock pollutant removal.

## 4. Conclusions

In summary, Ag-modified TiO_2_ thin films were successfully anchored on bamboo material through a facile hydrothermal process, followed by an Ag mirror reaction for photoactivated antifungal coating. Upon decorating Ag NPs on anatase TiO_2_ thin films, the composite films showed an enlarged optical response region and improved quantum efficiency. The antifungal test results show that the Ag/TiO_2_ composite films grown on the bamboo surface produced a synergistic antifungal mechanism compared with pure Ag NPs or anatase TiO_2_ film under both light and dark conditions. However, the antifungal activity of Ag/TiO_2_ composite films under visible light is superior to that in the dark, owing to the transfer of photoexcited electrons from Ag to TiO_2,_ their trapping on TiO_2_ under visible light illumination, and their release in the dark, which could give this photocatalyst a catalytic memory for producing ●O_2_^−^and ●OH radicals in the absence of light illumination. We believe that this discovery could open a door for the continuous removal of indoor air pollutants such as VOCs, bacteria, and fungus in the day and night time.

## Data Availability

Not applicable.

## Supplementary Materials

The following are available online at https://www.mdpi.com/article/10.3390/jof7080592/s1, Figure S1: Antifungal test. Figure S2: EDS spectrum of ATB-10. Inset images are corresponding elemental mapping results. Figure S3: XPS survey spectra of (a) original bamboo, (b) TB, and (c) ATB-10. Figure S4: SEM micrograph for a cross-sectional profile of the TiO_2_ thin film on bamboo substrate. Figure S5: XRD pattern of the as-prepared TB. Figure S6: High-resolution XPS spectrum of F 1s.
