# Supplementary material for: Visible-Light-Driven Ag-Modified TiO2 Thin Films Anchored on Bamboo Material with Antifungal Memory Activity against Aspergillus niger
Source: J Fungi (Basel). 2021 Jul 23;7(8):592. doi: 10.3390/jof7080592 (PMC8397055; doi:10.3390/jof7080592)
Supplement: Supplementary file 1 [file jof-07-00592-s001.zip › jof-1299045-supplementary.pdf]

Supplementary information

Visible-light-driven Ag-modified TiO_2 thin films anchored on bamboo material with antifungal memory activity against *Aspergillus niger*

Jingpeng Li[1,*], Rumin Ma[1], Zaixing Wu[1], Sheng He[1], Yuhe Chen[1], Ruihua Bai[1], Jin Wang[2,*]

[1] Key Laboratory of High Efficient Processing of Bamboo of Zhejiang Province, Engineering Technology Research Center for Building and Decorating Materials of Bamboo State Forestry Administration, China National Bamboo Research Center, Hangzhou 310012, PR China

[2] Key Lab of Biological and Chemical Utilizing of Forest Resources of Zhejiang Provincial, Zhejiang Forestry Academy, Hangzhou 310023, PR China

*Corresponding author: E-mail: lijp@caf.ac.cn (J.L.); whuwj@sina.com (J.W.)

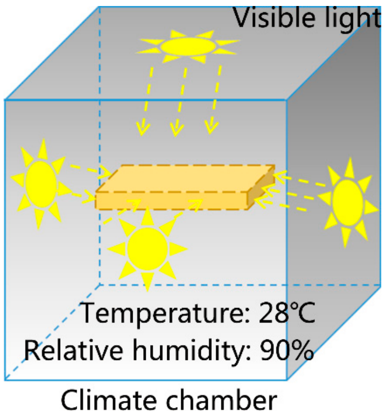

Figure S1. Antifungal test.

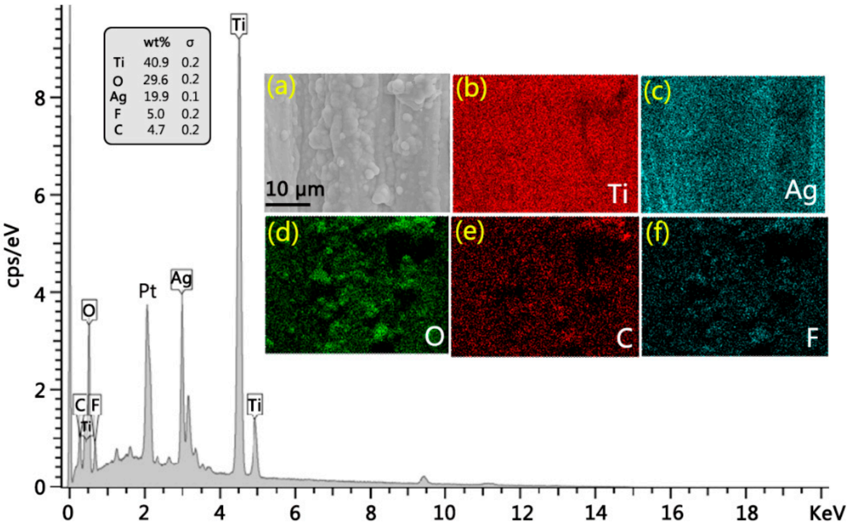

Figure S2. EDS spectrum of ATB-10. Inset images were corresponding elemental mapping results.

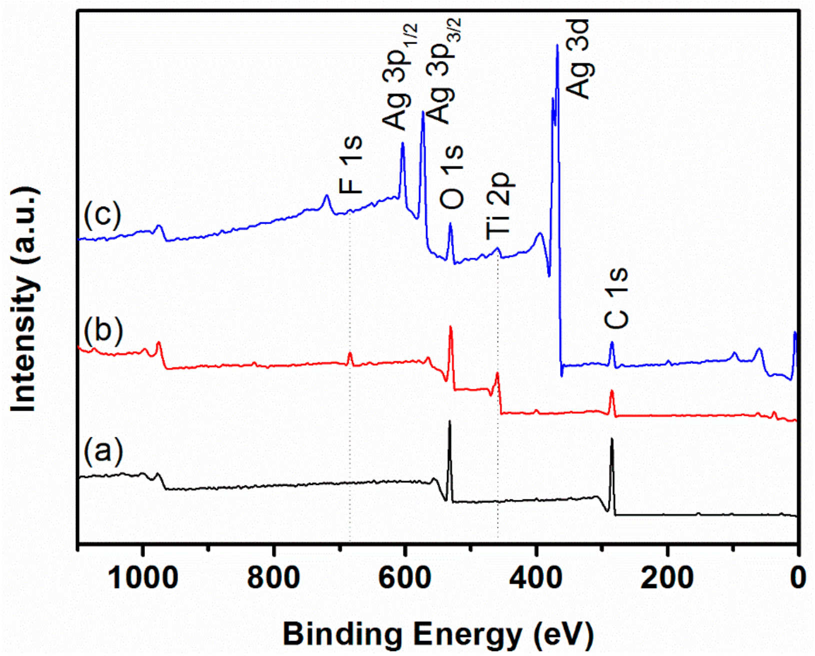

Figure S3. XPS survey spectra of (a) original bamboo, (b) TB, and (c) ATB-10.

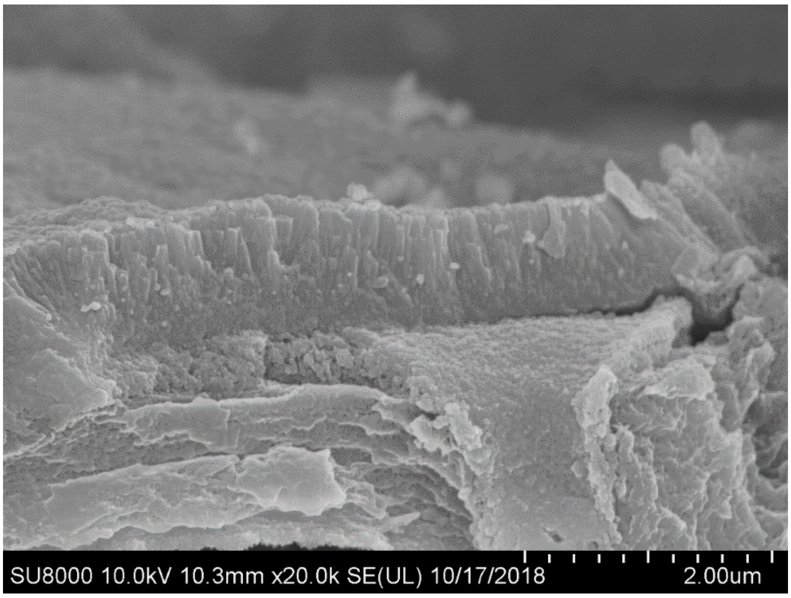

Figure S4. SEM micrograph for a cross-sectional profile of the TiO_2 thin film on bamboo substrate.

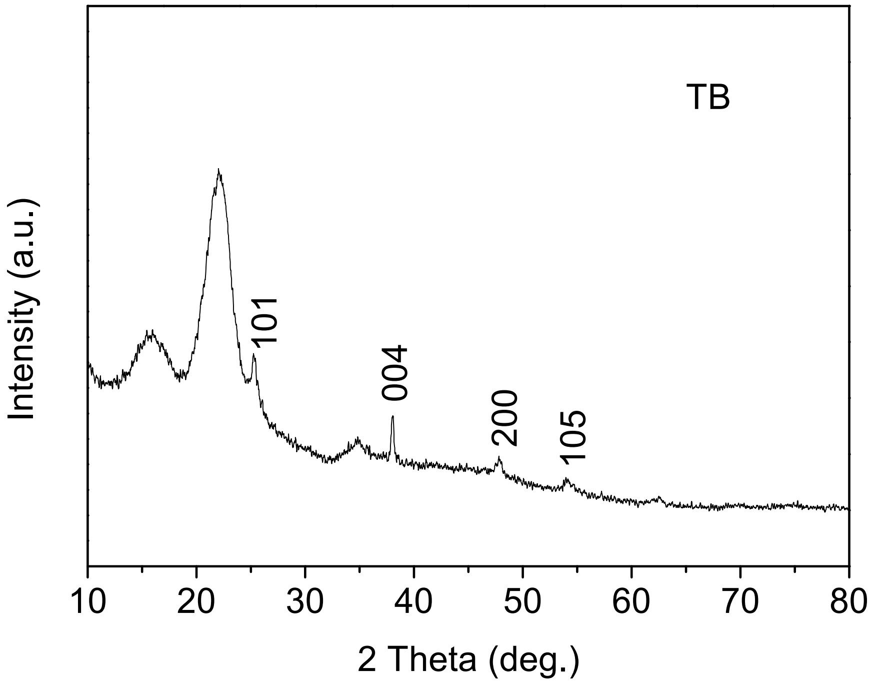

Figure S5. XRD pattern of the as-prepared TB.

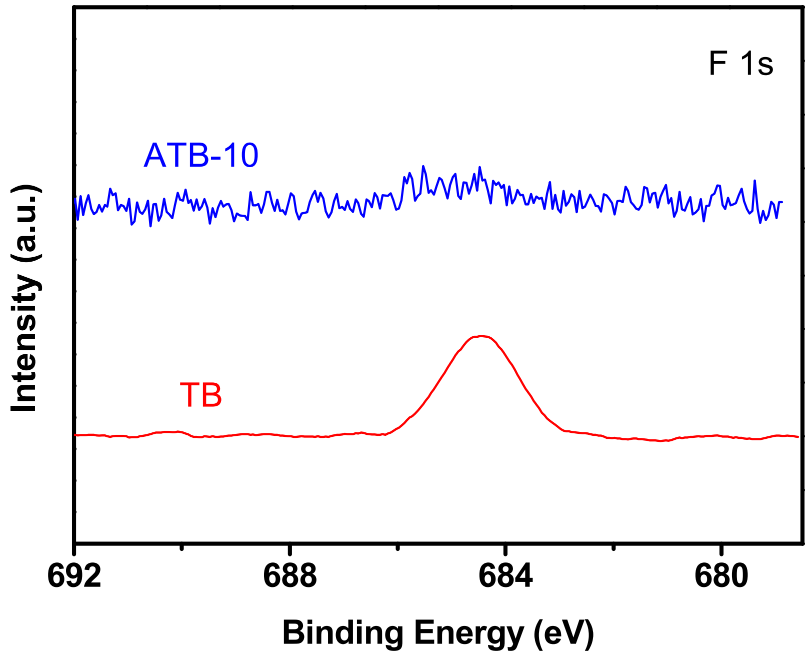

Figure S6. High resolution XPS spectrum of F 1s.
